# Radial endobronchial ultrasound in diagnosing peripheral lung lesions in a high tuberculosis setting

**DOI:** 10.1186/s12890-015-0089-9

**Published:** 2015-08-19

**Authors:** Adrian Chan, Anantham Devanand, Su Ying Low, Mariko Siyue Koh

**Affiliations:** Department of Respiratory and Critical Care Medicine, Singapore General Hospital, Singapore, Singapore; Duke- NUS Graduate Medical School, Singapore, Singapore

## Abstract

**Background:**

Current data for the utility of radial endobronchial ultrasound (EBUS) in investigating peripheral lung lesions (PLLs) has been restricted to populations with low pulmonary tuberculosis (TB) incidence. The aim of this study was to assess the diagnostic utility of radial EBUS with guide sheath in the diagnosis of peripheral lung lesions in Singapore, a high TB incidence setting.

**Methods:**

A post-hoc database analysis was performed. 123 consecutive patients with computed tomographic evidence of PLLs who underwent radial EBUS guided bronchoscopy were included.

**Results:**

The final diagnosis was malignancy in 76 cases and benign in 44 cases. Radial EBUS guided bronchoscopy had a sensitivity of 65.8 % for malignancy, positive predictive value of 100 %, negative predictive value of 62.9 %, and a diagnostic accuracy of 82.5 %. 22 patients had a final diagnosis of pulmonary TB. The diagnostic sensitivity for pulmonary TB was 77.3 %, with a positive predictive value of 100 %, negative predictive value of 95.2 % and a diagnostic accuracy of 95.8 %. Overall, 58.8 % of pulmonary TB cases relied on histology to make an early diagnosis.

**Conclusion:**

Radial EBUS guided bronchosopy is useful in investigating PLLs in a high TB incidence setting. Our data also suggests that radial EBUS is a more rapid diagnosis technique for tuberculous lesions.

## Background

Peripheral lung lesions (PLL) are defined as lesions that are not visualized within the bronchial tree during flexible bronchoscopy [1, 2]. Differential diagnoses can include both malignant causes and benign causes such as infection and inflammation. The diagnostic yield of flexible bronchoscopy in the biopsy of such lung lesions has been reported to be 54 % for malignant lesions and 41 % for benign lesions [3, 4]. To circumvent limitations of flexible bronchoscopy for such lesions, adjunct diagnostic tools have been proposed to guide bronchoscopic biopsies. Various studies have confirmed that radial EBUS is a modality that can improve diagnostic sensitivity [5–10]. The American College Of Chest Physicians Lung Cancer Guidelines has recommended using radial endobronchial ultrasound (EBUS) as an adjunct imaging modality for patients with peripheral lung nodules, where expertise and equipment are available. It can be used especially in cases where tissue diagnosis is required due to the uncertainty of diagnosis or poor surgical candidacy (Grade 1C evidence) [11]. Such guidelines are targeted at populations of an intermediate probability of lung cancer depending on clinical history, radiological stability and CT signs of malignancy such as size, spiculated borders, and absence of calcification.

However, pulmonary tuberculosis (TB) can also present as a peripheral lung lesion with varying disease activity: active infection, tuberculous granulomas, or inflammatory scars. In populations where tuberculosis is endemic, benign-looking lesions (based on clinical history and radiological characteristics) cannot always be managed with radiological surveillance because of both therapeutic implications and public health reasons. Delayed diagnosis and empiric tuberculosis treatment in an era of drug resistance will undermine the global control of TB [12, 13]. Percutaneous lung biopsy and surgical resection are options that offer a high diagnostic yield, but these will expose patients with a potentially curable infection to unnecessary procedural risks.

The diagnostic yield of conventional (transbronchial biopsy) TBB for pulmonary TB has been reported to be 55–75.8 % [14, 15]. The current literature on radial EBUS is limited mostly to populations with high lung cancer prevalence and there is limited data on the role of radial EBUS guided bronchoscopic biopsy for PLLs in populations with a high incidence of pulmonary TB [9–11]. We aimed to evaluate the diagnostic yield of radial EBUS-guided sampling in PPLs for both malignant and benign lesions (including TB).

## Methods

### Study design and setting

This is a retrospective database review of an established database in Singapore General Hospital, a 1700-bedded university-affiliated tertiary hospital. A database of all adult consecutive patients at our hospital who consented for and underwent flexible bronchoscopy using radial EBUS was established in August 2008. This project was supported by government funding from the Ministry of Health, Singapore (under Health Services Development Project). As such, the project was closely audited by the funding agency and the Singapore General Hospital Quality Management Department. Therefore, completeness of data collection, diagnostic yield, technical details and complications were ensured. The study was approved by SingHealth Centralised Institutional Review Board (CIRB: 2008/458/B).

### Study population

All consecutive patients who had PPLs and had undergone radial EBUS were considered for inclusion. Exclusion criteria were: 1) CT findings with any of the following: PPL < 1 cm diameter, endobronchial lesions, airway narrowing, pure ‘ground-glass’ appearance, or absence of CT bronchus sign, and 2) Presence of a submucosal lesion seen during flexible bronchoscopy. The study period was between August 2008 and December 2011 and data for included patients were reviewed retrospectively.

### Study procedures

Bronchoscopy was performed under moderate sedation using a combination of midazolam and fentanyl. The pre-test probability of pulmonary TB was considered high if there was fever and sputum production clinically and if there was cavitation radiologically. All these patients had negative acid-fast bacilli smears prior to endoscopy.

During the procedure, the bronchoscope was maneuvered to the suspected sub-segmental airway and BAL was first performed. This was followed by insertion of a 20-MHz radial EBUS probe (UM-S20-20R; Olympus, Tokyo, Japan) with an external diameter of 2.2 mm via a guide-sheath through the 2.8 mm working channel of the flexible bronchoscope (Exera BF-1 T260; Olympus, Tokyo, Japan) with an outer diameter of 6.0 mm. The lesion was localized when the ‘snow-storm’ ultrasound appearance of aerated-normal lung was replaced with a soft tissue density on the processor monitor (EU-ME1; Olympus, Tokyo, Japan). The radial probe was adjusted until the probe was within the lesion. This was continued until all visible sub-segments were explored. Ultimately if the radial probe could not be placed within the lesion, the best CT-bronchoscopic correlation was used. Then, the probe was withdrawn leaving the guide-sheath in situ. Aliquots of 20 ml of normal saline solution were instilled and retrieved immediately with negative suction pressure that was adjusted to avoid airway collapse. A total of 100 to 200 ml of normal saline was instilled in each patient. Forceps biopsies were then taken under fluoroscopic guidance. Fluoroscopy served the dual purposes of ensuring that the guide-sheath was not dislodged and the forceps were closed at a safe distance from the pleura. Aliquots from the pooled BAL sample were sent for Gram stain, acid fast bacilli (AFB) smear, microbiologic (including tuberculous) culture and cytologic examination; forceps biopsies were sent for histopathology and tuberculous cultures.

Malignant PPLs was diagnosed based on histological evidence of malignancy obtained from bronchoscopic biopsies. A diagnosis of tuberculosis was derived based on the following outcomes: a) presence of positive cultures or b) demonstration of necrotizing granulomatous inflammation on histology with or without positive microbiology or c) clinical suspicion of TB and response to empirical anti-tuberculous treatment with radiological resolution of PPL [16]. All patients with non-diagnostic bronchoscopy were either subjected to an alternative biopsy (usually CT-guided or surgical biopsy) or followed up with a combination of radiology and clinical surveillance for a minimum of 12 months, depending on the managing physician’s discretion. When an alternative diagnosis was established, these cases were designated as false negatives. If the patient showed both clinical and radiological stability, then the lesion was considered likely to be pulmonary scar tissue and designated as a true negative.

### Statistical analysis

Statistical analysis was performed using a statistical package for social sciences software (Version 21.0). Confidence intervals of 95 % were reported, and all tests were 2-sided. Continuous variables were expressed as mean ± standard deviation, and comparisons were analysed with t-tests. Outcomes for categorical variables were analysed using chi-square test or Fisher’s exact test. Sensitivity, specificity, predictive values and accuracy were calculated based on standard definitions. *p* < 0.05 was regarded as being statistically significant.

## Results

### Patient characteristics

Overall, 123 patients underwent bronchoscopy with radial EBUS guidance. 3 patients were excluded from analysis as the final diagnosis could not be determined due to death (2 patients) or loss to follow-up (1 patient) [Table 1]. The mean age of the patients was 62.6 ± 12.6 years and 60 % were males. The median number of forcep-biopsies performed was 5 (range 1–13). The median midazolam dose was 3.0 mg (range 0–10 mg) and the median fentanyl dose was 50.0 mcg (range 0–200 mcg). Patients diagnosed with malignant lesions were significantly older than those with benign lesions (mean age: 65.1 ± 10.3 vs. 58.2 ± 15.0 years, *p* = 0.004).

**Table 1:** Demographics, clinical-radiological data and bronchoscopic results of malignant and benign peripheral lung lesions

	Malignant		Benign		*p*-value
Demographics					
Subjects (n)	76		44		
Age	65.1 ± 10.3		58.2 ± 15.0		0.004
Male gender (%)	60.5		70.5		0.326
Smoking (%)	44.6		33.3		0.247
Diabetes mellitus (%)	21.6		14.3		0.461
Presenting symptoms (n)					
Cough		27		14	0.841
Dyspnoea		14		1	0.037
Weight Loss		12		4	0.440
Hemoptysis		5		4	0.721
Fever		2		5	0.097
Asymptomatic		23		16	0.442
Size					
Mean (mm)	26 ± 12		24 ± 13		0.357
≤20 mm	28 (36.0)		21 (47.7)		
>20 mm	48 (64.0)		23 (52.3)		
Common radiologic characteristics					
Spiculation/Lobulation		44 (57.9)		10 (23.8)	<0.001
Cavitation		4 (5.3)		13 (31.0)	<0.001
Ground gass/Semisolid		4 (5.3)		3 (7.1)	0.698
Appearance					
Tree-in-bud changes		1 (1.3)		6 (13.6)	0.009
Distance of lesion from pleura (mm)	17 ± 13		16 ± 14		0.606
Lobar Position (n)					0.582
Right upper lobe	20		12		
Right middle lobe	6		5		
Right lower lobe	20		8		
Left upper lobe	28		14		
Left lower lobe	2		5		
Final diagnosis					
	Non-small cell lung cancer	63 (82.9)	Pulmonary tuberculosis	22 (50.0)	
			Pneumonia	15 (34.1)	
	Small cell lung cancer	2 (2.6)	Other diagnoses		
	Pulmonary metastases	10 (13.2)	Organising pneumonia	5 (11.4)	
	Bronchial carcinoid	1 (1.3)	Scarring	2 (4.6)	
Cases diagnosed by radial EBUS	Non-small cell lung cancer	45 (59.2)	Pulmonary tuberculosis	17 (38.6)	
			Pneumonia	12 (27.3)	
	Small cell lung cancer	2 (2.6)	Other diagnoses		
	Pulmonary metastases	2 (2.6)	Organising pneumonia	2 (4.5)	
	Bronchial carcinoid	1 (1.3)	Scarring	2 (4.5)	
Yield based on known location of probe					
Within lesion	49 (71.6)		29 (78.9)		
Adjacent to lesion	16 (23.9)		6 (15.8)		
Outside lesion	3 (4.5)		2 (5.3)		

Data presented as mean + SD and n (%), unless otherwise stated

### Diagnostic yield and outcomes

The final diagnosis was malignancy in 76 cases, giving an incidence of 62.5 %. Radial EBUS for malignant lesions had a sensitivity of 65.8 % (95 % CI: 53.9–76.0), positive predictive value of 100 % (95 % CI: 91.1–100), negative predictive value of 62.9 % (95 % CI: 50.4–73.9), and an overall diagnostic accuracy of 82.5 % [Table 2]. There was no difference for yield between malignant and non-malignant lesions (*p* = 0.308). 10 lesions were diagnosed to be pulmonary metastases; the bronchoscopic diagnostic yield for such lesions was only 20 %. 21 of 26 patients with non-diagnostic EBUS-guided sampling underwent further invasive investigations to confirm the diagnosis of malignancy [Table 3].

**Table 2:** Histologic and/or microbiologic results obtained from specimens derived from EBUS-guided sampling as compared to the final diagnosis of malignancy

	Final diagnosis of malignancy	Final diagnosis of benign lesion	Total
EBUS sampling positive for cancer	50	0	50
EBUS sampling negative for cancer	26	44	70
Total	76	44	120

**Table 3:** Evaluation of patients who had non-diagnostic radial-EBUS guided bronchoscopy

Malignant	n = 26
Final method of diagnosis	
Percutaneous lung biopsy	12
Surgical lung biopsy	9
Radiologic progression of pulmonary metastases	2
Transbronchial needle aspiration of associated lymph node metastases	1
Repeat bronchoscopic biopsy with radial EBUS guidance	1
Response to chemotherapy	1
Benign	n = 11
Tuberculosis	
Final method of diagnosis	
Responded to anti-tuberculous therapy	3
Percutaneous lung biopsy	1
Surgical lung biopsy	1
Infection	
Final method of diagnosis	
Responded to anti-microbial therapy	3
Organising pneumonia	
Final method of diagnosis	
Repeat bronchoscopic biopsy with radial EBUS	2
By different bronchoscopist	
Scarring	
Final method of diagnosis	
Interval stability on repeat imaging (up to 1 year)	1

22 patients in our study group had a final diagnosis of pulmonary TB. Radial EBUS guided bronchoscopy successfully diagnosed pulmonary TB in 17 out of 22 cases, providing a diagnostic sensitivity of 77.3 % (95 % CI: 54.2–91.3), positive predictive value of 100 % (95 % CI: 77.1–100), negative predictive value of 95.1 % (95 % CI: 88.5–98.2) and a diagnostic accuracy of 95.8 % [Table 4]. Mycobacterial culture was positive in 15 of 17 (88.2 %) cases. These were obtained from BAL in all cases, whilst 6 cases yielded a positive culture from transbronchial biopsies as well. 10 of 22 patients demonstrated necrotizing granulomatous inflammation on their TBBs. In this subgroup with positive histology, all patients had negative AFB smears on bronchoscopic samples, and 2 patients had negative tuberculous cultures. In patients with negative histologic findings on biopsies, the main pathologic findings were chronic inflammatory changes without granulomatous inflammation (11 patients) or necrotic material (1 patient).

**Table 4:** Histologic and/or microbiologic results obtained from specimens derived from EBUS-guided sampling as compared to the final diagnosis of tuberculosis

	Final diagnosis of tuberculosis	Not tuberculosis	Total
EBUS sampling positive for tuberculosis	17	0	17
EBUS sampling negative for tuberculosis	5	98	103
Total	22	98	120

Data for ultrasound probe location was available for 105 patients. The probe was within the lesion in 78 patients (74.3 %), adjacent to the lesion in 22 patients (21.0 %), and outside of the lesion in 5 patients (4.7 %). Yield was significantly higher when the probe was positioned within the lesion (78.2 %) than when the probe was adjacent to or outside the lesion (44.4 %) (*p* = 0.001).

Mean lesion diameter was 25.6 ± 12.4 mm, with 71 patients (59.7 %) having lesions greater than 20 mm diameter. Mean lesion diameter was similar for malignant lesions (26 ± 12 mm) when compared to benign lesions (24 ± 13 mm) (*p* = 0.357). There was no significant difference in the yield for lesions greater than 20 mm diameter as compared to lesions ≤ 20 mm in diameter (67.6 % vs.70.8 %) (*p* = 0.840). Lobar location of the lesion also did not affect diagnostic outcome (*p* = 0.590). Mean lesion distance from pleura was 16.8 ± 13.5 mm.

Overall complication rate was 6.4 % [Table 5]. Seven patients (5.6 %) had bleeding that required cold saline or topical adrenaline for haemostasis and 1 patient (0.8 %) suffered a pneumothorax that was managed conservatively without requiring chest drainage. All complications were self-limited and no patient required escalation in the level of care.

**Table 5:** Complications arising from radial-EBUS guided sampling

	Malignant	Benign
Bleeding	5	2
Pneumothorax	0	1
Death	0	0

## Discussion

Although previous studies that investigated PLLs using bronchoscopy with radial EBUS guidance have demonstrated a high sensitivity for the detection of malignant lesions, it is worth noting that majority of the reports came from populations with high incidence of malignancy [17–20]. Lai et al. had a pulmonary TB incidence of 23.5 %, and reported a sensitivity of 55 % for tuberculous lesions diagnosed via conventional bronchoscopy and fluoroscopy [15]. In comparison, the incidence of pulmonary TB in our setting was 18.3 %. The addition of radial EBUS with guide-sheath increased the diagnostic sensitivity to 77.3 %, consistent with previous studies that highlighted an increased yield could be obtained when these 2 techniques are combined [5, 21, 22].

Another important finding from our study is that radial EBUS guided TBBs provided a more rapid diagnosis via histology in nearly half of patients with pulmonary TB, given their initial negative smear microscopy. Tuberculosis remains a global epidemic, and the ability to diagnose pulmonary TB early is important from a clinical and public health perspective. In 2011, there were an estimated 8.7 million new cases of TB and 1.4 million deaths from TB [23]. Around 3 million new cases of TB cases are diagnosed in South-East Asia each year, and this region accounts for 39 % of the global burden of TB [23]. Singapore, with an incidence of pulmonary tuberculosis of 50 per 100,000, is situated in a tuberculosis endemic region and offers a setting to further understand EBUS diagnostic yields for both malignant and tuberculous diseases [24]. Early diagnosis and appropriate treatment of infectious patients with pulmonary TB are important steps to reduce transmission of *Mycobacterium Tuberculosis* and to achieve disease elimination [23]. Radial EBUS aided in obtaining histological diagnosis consistent with pulmonary TB infection in 58.8 % of our pulmonary TB patients thus enabling pulmonologists to diagnose the disease and start anti-tuberculous treatment earlier. However, histology does not replace mycobacterial cultures, which is the “gold standard” in diagnosis of pulmonary TB that gives additional important information on sensitivity to anti-tuberculous medications. Indeed, this is very important in the era of drug-resistant TB [23].

Our study demonstrated a low complication rate (6.4 %) comparable to previously reported safety data of radial EBUS (0–7.4 %) [9]. This is much lower compared to the generally quoted complication rate from percutaneous lung biopsy (15–25 %) [25, 26] with similar yield of 68.6 % for pulmonary TB [27]. The ability to diagnose pulmonary TB earlier with histology and low complication rate highlights the potential of radial EBUS as the preferred first step in an algorithm to evaluate PLLs when the pre-test probability of tuberculosis and malignancy are similarly high or for high risk patients who may not be able to tolerate the complication of pneumothorax (for example, patients with emphysema or chronic obstructive pulmonary disease). It is also noted that adding guide sheaths to the procedure does not worsen the safety profile and its use should be considered for sampling [28].

We postulate that our higher yield for benign lesions could be due to such lesions having surrounding inflammation with ill-defined borders - or in the case of pulmonary TB, associated consolidation, centrilobular nodules or tree-in-bud changes. In our group of pulmonary TB patients, the following associated radiologic characteristics were observed in addition to the PLLs: cavitation (9 lesions), scarring (3 lesions) and ground glass opacities (2 lesions). Another possible reason could be that benign lesions are less likely to cause airway distortion, in contrast to malignant lesions that have been recognized to directly compress and narrow the bronchus or indirectly narrow the proximal bronchial tree due to enlarged peribronchial or submucosal lymph nodes [29].

We report a sensitivity of 65.8 % for all malignant lesions, which is similar to yields obtained from previous radial EBUS studies [9]. However, our diagnostic yield for pulmonary metastases was much lower (20 %). This can be explained by spread of pulmonary metastases being haematogenous and not bronchial; hence visualization and diagnostic yield of such lesions by EBUS may be lower. This is supported by our observation that we could place our probe within the metastatic lesion in only 30 % of cases. Percutaneous lung biopsy might be more appropriate in such cases.

We identified several limitations in our study. Firstly, during the time of study, the use of nucleic acid amplification (NAA) tests as well as molecular tests to detect TB and mutations conferring rifampicin resistance (eg Xpert MTB/RIF) were not widely available for routine clinical use in our institution. The advantage of NAA is its ability to provide results within 24–48 h, compared to 3–5 days for histology and 2–6 weeks for TB cultures [30]. NAA testing has a high positive predictive value (>95 %) in AFB smear-positive sputum specimens (especially relevant in settings in which non-tuberculous mycobacteria are common). It can confirm the presence of Mycobacteria tuberculosis in 62.2–79.3 % of AFB smear-negative, culture-positive specimens [31, 32]. In our study group, all our pulmonary TB patients were sputum AFB smear negative. Given the low sensitivity of NAA in such patients and the fact that histology is necessary for the diagnosis of malignancy, these tests were unlikely to have made a huge impact in our clinical decision to perform bronchoscopy and radial EBUS. Secondly, being a retrospective analysis, selection bias could not be excluded. However, this was minimized by including all consecutive patients who received radial EBUS guidance in our institution. This meant that we could not apply clinical-radiologic models in predicting the probability of malignancy or infection prior to histologic investigation, which may subsequently influence the diagnostic yield of EBUS-TBB. We did not include a control group of patients with PPLs who underwent bronchoscopy with conventional fluoroscopic biopsy for comparison of diagnostic yields, as such patients should have been considered for an alternative procedure given the published data on low yields from conventional biopsies. Unlike previous reports where EBUS bronchoscopies were performed by selected few experts, radial EBUS bronchoscopies were performed by all 8 pulmonologists in our institution, hence variability in endoscopic competency may have affected the yield. Nevertheless, our hospital has a high bronchoscopic load with >1000 bronchoscopies performed every year and all pulmonologists were experienced bronchoscopists trained in EBUS at the same time. Therefore, endoscopic competency would not be a major factor in influencing the yield. Furthermore, this provides ‘real-world’ experience rather than procedures performed by selected specialized experts. It has been reported that performing trans-bronchial needle aspiration with radial EBUS guidance further improves the diagnostic yield [33]. This technique was not available to our center at the time of the study and could be evaluated in future studies. It was previously reported that EBUS-guided biopsies without the use of fluoroscopy had limited benefit for diagnosing smaller (≤20 mm) lesions [17]. For our study, we elected to combine both EBUS and fluoroscopy for optimal guidance of the forceps. Thus, the exact usefulness of EBUS may have been overestimated in our study especially for smaller lesions. This may also account for our similar diagnostic yields for smaller and larger lesions. Ideally, the gold standard for final diagnosis should be histologic or microbial confirmation of all PPLs. However, the risk-benefit ratio of multiple diagnostic procedures was considered and we included patients who we were confident of the final diagnosis based on appropriate observation or treatment.

Limitations notwithstanding, the findings from our study have several clinical implications. Taking into account the high diagnostic accuracy for both malignant and benign lesions as well as the excellent safety profile of radial EBUS guided TBBs, we suggest using this as the first modality to investigate PLLs in which clinical characteristics and sputum studies remain inconclusive. This may in turn reduce morbidity and healthcare costs for patients who ultimately have benign lesions. Secondly, in cases where pre-test probability of pulmonary TB is high, whilst it is sufficient to obtain an eventual microbiologic diagnosis with BAL alone, the added benefit of a more rapid histologic diagnosis with radial EBUS guided TBB should be considered [34]. To validate these recommendations, prospective studies should be conducted in populations with a similar prevalence of pulmonary TB. This will allow for further analysis of the test performance of radial EBUS of pulmonary TB with the aim of creating a clear management algorithm of PLLs.

## Conclusion

In our population with a high incidence of pulmonary TB, it is important to review the clinical and radiologic characteristics of PLLs before deciding on choice of investigation for histologic diagnosis. Where clinical suspicion of malignancy is indeterminate or when pulmonary tuberculosis is suspected following initial negative sputum studies, radial EBUS is to be considered as a viable first step in the evaluation of PLLs. The additional yield, faster time to diagnosis and favourable safety profile of radial EBUS are the positive factors contributing to our recommendation.

## Abbreviations

AFB: Acid fast bacilli
BAL: Bronchoalveolar lavage
CT: Computed tomography
EBUS: Endobronchial ultrasound
NAA: Nucleic acid amplification
PLL: Peripheral lung lesion
TB: Tuberculosis
TBB: Transbronchial lung biopsies
